# Tuberculosis: Medico-Legal Aspects

**DOI:** 10.4084/MJHID.2014.033

**Published:** 2014-04-19

**Authors:** G. Vetrugno, F. De-Giorgio, F. D’Alessandro, I. Scafetta, F. Berloco, D. Buonsenso, F. Abbate, G. Scalise, V.L. Pascali, P Valentini

**Affiliations:** 1 Direzione Rischio Clinico e Igiene, Policlinico Universitario “A. Gemelli”, L.go “A. Gemelli”, 8, Roma.; 2 Istituto di Sanità Pubblica – Università Cattolica del S. Cuore, Roma.; 3 Centro Studi “Federico Stella” – Università Cattolica del S. Cuore, Milano.; 4 Dipartimento per la tutela della salute della donna, della vita nascente, del bambino e dell’adolescente – Policlinico Universitario “A. Gemelli”.; 5 Avvocato del Foro di Roma.

## Abstract

Tuberculosis is a diffusive infectious disease whose typical behaviour differentiates it from other infectious diseases spread by human-to-human transmission (flu, chicken pox, cholera, etc.) that follow a classic epidemic pattern. Indeed, in the presence of a known source of Koch bacilli that is capable of spreading the bacteria by air, not all exposed individuals inhale the bacteria, not all those who inhale them absorb them, not all those who absorb the bacteria are unable to eliminate them, not all who are able to eliminate them do so using delayed hypersensitivity, not all those who react with delayed hypersensitivity suffer lasting tissue damage (among other things, minor), not all who suffer tissue damage have anatomical sequelae, and not all those who have anatomical sequelae, however minimal, become carriers of bacilli in the latent period.

The vast majority (90–95%) of the latter – which are in any case a portion, not the totality of those exposed – remain asymptomatic throughout their lives and never develop active tuberculosis.

Based on these biological characteristics and the legal concepts of “epidemic” and “disease,” it becomes highly problematic, if not impossible, to assert both that tuberculosis can cause events of sufficient magnitude to be associated with the crime of “epidemic,” and that the mere diagnosis of a latent tuberculosis infection is sufficient to assume the presence of an illness legally prosecutable in criminal proceedings or a disability prosecutable in civil proceedings.

Furthermore, clinically apparent tuberculosis is a temporarily—and in some cases permanently—disabling condition, and in certain work environments, even with the difficulties caused by the lack of available effective diagnostic tools and the insidious behaviour of the disease in the early stages, targeted monitoring to identify other persons who may become ill is appropriate.

## Introduction

In the national legal systems of modern liberal states, health as an individual right has been historically fundamentally perceived as an issue of domestic and international public order.

Hygiene and prevention policies designed to contain the epidemiological events that resulted in mass deaths in European countries were implemented and exemplified a more general policy of national control. With respect to public order, these policies sometimes led to concrete measures that quite often had elements of repression.

Closing off cities and quarantining them to prevent the spread of epidemics is the most concrete historical example of such measures, though even in our current globalised world, risks and emergencies must be addressed and resolved through measures that often include forms of isolation, both in a national and European Union context, and internationally (e.g., mad cow disease, avian flu, swine flu etc.).^1^

In this historical context, during which the goal was mainly to develop procedures to protect domestic public order, with no importance given to health as an individual right, it is understandable how states made extensive use of criminal penalties as tools of repression. Together with administrative public health measures, local authorities (mayors, local police, etc.), not only created categories of offenses to shelter the community from infection by so-called “disease spreaders” but also developed a concept of disease that differs from the one traditionally used in medical practice.

Not that the scenario has particularly changed in recent years, since, on the contrary, with the appearance of the so-called risk society^2^ and with the acceptance of the fallibility of science in the eyes of the law,^3^ the introduction of the precautionary principle and its varying application in international law^4^ have consolidated the approach under which health problems are brought back within the sphere of safety or public order, i.e., as a public health issue viewed as an emergency of international and domestic public policy.

Moreover, to cite the distinguished scholar Klaus Lüderssen of Frankfurt, when problems are difficult and complex, civil law and administrative law “pass the baton” over to criminal law,^5^ reserving only corollary issues, including compensation and social insurance issues.

Criminal law also has mechanisms that have been oiled over time and which, precisely because of their extensibility, are believed to be applicable to control the spread of disease in communities: these are the crimes of murder, bodily injury and criminal dissemination of an epidemic.

But however, when evaluating the implementation of these three charges in actual cases that come before criminal courts, one quickly realises the substantial ineffectiveness of such measures – designed to punish those responsible for a death or injury and not to stop the outbreak of contagion – for addressing the need to protect the health of the community and the need to identify those responsible beyond a reasonable doubt.

Within this framework lies the scope of this article, the medico-legal implications of tuberculosis in criminal, civil and administrative law. Tuberculosis, a historical infectious disease, while not sharing the contagion characteristics of other more typically epidemic diseases such as influenza, cholera and the now-defunct smallpox, continues to fuel unrest in communities, partly because of the media’s behaviour, which is primarily focused on sensationalism rather than broadcasting fact-based news. Today, immigrants in particular are chillingly viewed as “disease spreaders” and scapegoated, as the Landsknecht mercenaries before them, who were blamed for the 1630 plague in Milan.^6^

## Can Tuberculosis (TB) Cause an Epidemic Prosecutable Under Criminal Law?

The concept of epidemic in medical science differs from the definition developed in criminal law.

In medicine, an epidemic is an event that evolves suddenly and affects more people in a particular area than would normally be expected.^7^

In criminal law, on the other hand, for an epidemic to be a crime there must be proof of an unexpected and mass onset of an infectious human disease, capable of spreading quickly within a particular time and space and potentially affecting a significant, indeterminable number of people.

This particular crime, which is dealt with in article 438 of the Italian Penal Code, is designated as one of causing criminal harm, qualified by the existence of a real danger to public health.^8^ In fact, the particular semantics of the word epidemic, when it is used, infers that in order to confirm that an epidemic exists, the expositor must immediately verify two predicaments. The first is a significant succession of occurrences detrimental to the health of identifiable individuals that can be legally determined as murder or harmful, and the second is the potentially unstoppable spread of a disease that, on the basis of similar situations in the past, would endanger the health of a large but indeterminate number of people in the community. In these circumstances, “an epidemic is not just any infectious disease, but is one that given the ease with which its germs can be propagated, puts the health of a great many people in any single context at risk”.^9^

In other words, in order to speak of an epidemic in the legal sense, the situation on the ground must present the following characteristics:

1. The existence of an infectious disease that spreads quickly and in such a way that anyone that has been infected is instantly capable of transmitting the germs to others. As a result, this disease is capable of infecting an indeterminable number of people, in a brief period of time, in what could be a larger or smaller area such as a town, a city or even a region.
2. Measures taken by the Public Health authorities (the mayor, health workers etc.) have not succeeded in quickly containing the spread of the disease.

The actual number of people infected is irrelevant to the determination of whether there is an epidemic underway. It does have a bearing on the process of identifying a person stricken with an infectious disease as someone who is a looming threat to an indeterminable number of potential victims, within an extended community, in an uncontrollable and non-discriminating situation that agencies and Public Health Authorities cannot manage.

Insomuch as, from the perspective of criminal legislators, the negative value conveyed by the crime of causing an epidemic transcends that of single harmful acts such as murder and criminal harm, and provides a safeguarding mechanism centred entirely on potential victims (those at risk of secondary infection) rather than existing victims (those with primary infection). The severity of the crime of causing a risk to public safety, therefore, means that when ascertaining the existence of a criminally relevant epidemic already underway,^10^ and knowing it has already infected many people and could infect many more in the future, especially as the responsible health authorities are proving incapable of bringing it under control, it is essential to identify the characteristics of its diffusion and the likelihood of not being able to control its further spread.

This last point is particularly important, because the immediate implementation of effective containment measures by Public Health Authorities and the incapacity of the disease to spread to a large number of people exclude the possibility, for the prosecutor, to charge the crime of causing an epidemic.

The logical consequence that can be inferred from this then, is that any outbreaks of micro-epidemics (i.e., localised situations or those contained within specific communities) cannot be part of the concept of the crime of causing an epidemic.

In the same way, incidents of infectious diseases that occur at the same time, harming patients admitted to the same hospital, but that do not spread outside the structure to the rest of the population, do not seem to comply with the definition of the crime in question.

Similarly, episodes of infectious diseases caused by a single germ infecting hospital patients at the same time, but not spread outside the hospital among the general population, do not meet the requirements for the crime in question.

This explains why, for example, an Italian court considered the accusations groundless in a case involving the death of sixteen infants hospitalised in the nursery ward of a paediatric clinic as a result of infection with Salmonella Wien. The disease had developed within a closed environment and did not have the magnitude and diffusion sufficient to constitute a danger to the health of an indefinite number of people (additionally, the outbreak was contained and fought with no further danger of exterior transmission).^11^

Further emphasising the substantial gap between the medical-scientific meaning of epidemic and the meaning used in criminal law is the consideration that charges of epidemic dissemination can be upheld only in cases of simultaneous or rapid onset of a disease suspected to be an epidemic, because the lack of this condition is indicative of poor transmission of pathogens still capable of inter-human transmission.

One can well understand, therefore, how within this interpretative framework, the epidemiological profile of tuberculosis is badly suited to cases of criminal epidemic dissemination.

The natural history of the disease, which has been known for centuries, reveals that tuberculosis is indeed a diffusive infectious disease that behaves in a manner significantly different from other infectious diseases such as influenza, chickenpox and others that have typically and concretely proven their ability to individually trigger real epidemiological outbreaks.

The reason for this lies in the fact that not all those who come into contact with the bacteria responsible for tuberculosis become infected. The most likely rate of infection is 20–30%,^12^ and only a fraction of those infected – approximately 5–10% – go on to develop the disease (after some time) and the ability to infect others.

In practice, all those who are actually infected with the bacteria are unable to pass it on to others if they have not themselves also developed the disease in its so-called active form. This makes it difficult to meet the concentrated space and time conditions required for charges of criminal epidemic dissemination.

In fact, because of its progression and development characteristics, it is almost impossible for a disease such as tuberculosis to spread across a population in such a way as to make a large number of people ill (and not merely infected ) within a given time frame.

Of note is the historical case of the mass death of children in Lübeck in 1929–1930. The medico-legal experts of the time were able to demonstrate that it occurred as a result of vaccinations carried out using a batch of Calmette-Guerin bacilli accidentally contaminated by live Kochbacilli.^13^ Although this event has the characteristics of an epidemic from a strictly medical point of view, it could not be classified as an epidemic in terms of criminal law, precisely because it lacked the requirement of further spread of the disease beyond the circle of those directly and immediately infected. Furthermore, the very rapid development of the disease in that environment was made possible by the particular mode by which the germ was introduced to a small, at-risk population.

Moreover, the most recent observations of hospital settings demonstrate that even in the presence of an infectious source with the capacity to spread the bacteria in a manner generally favourable to epidemic events (i.e., by air and over a protracted period of time), exposure of a significant number of people did not result in subsequent illness.

In December 2003, in a large New York hospital, a registered nurse working in a maternity ward and nursery, whose status as a carrier of tuberculosis infection for the past eleven years was known, was diagnosed as suffering from pulmonary tuberculosis. The nurse had begun to show the first symptoms of the disease in September 2003, but continued to work in part because of negative results to the diagnostic tests that had been performed.

Health authorities identified the nurse’s window of infectivity as spanning the period between 1 September and 29 November 2003, and initiated an epidemiological investigation within this window, without detecting the disease among those exposed.^14^

In August 2006, in a hospital near Kyoto, Japan, another registered nurse who worked at the hospital maternity ward and nursery, was found to be suffering from pulmonary tuberculosis. Her most significant symptoms (starting with productive cough) began in April 2006, and the nurse had continued to work due to the negative results of the first clinical and diagnostic evaluation.

Also in this case health authorities identified the infection period of the nurse as the interval between the onset of symptoms and the date of definitive diagnosis, i.e. between April and August 2006; after examining subjects who came in contact with the nurse within the window of infectivity, no additional diseases were detected.^15^

These examples reveal how, first, it is extremely difficult to intercept active tuberculosis in a timely manner, because delayed diagnosis is a constant of the disease and can exceed two months.^16^ Second, it is particularly difficult to identify even the onset of the active stage, so much so that clinical guidelines provide empirical criteria that place the beginning of this stage at three months prior to diagnosis of TB, sometimes stressing the importance of medical history data and sometimes the significance of X-ray data.^17^ This not only diminishes any attempt to determine the start of the infectious stage but also prevents identification of an estimated initial time of a hypothetical epidemic episode.

In the last century, tuberculosis was endemic in Italy and was very much present in everyday life, not only for doctors. Not surprisingly, therefore, a distinguished Italian jurist of the time, in his momentous Treatise on Italian Criminal Law, was able to reasonably rule out that tuberculosis could ever become an epidemic.^18^

In conclusion, because in legal terms the concept of epidemic requires not only a phenomenon of substantial magnitude, defined as an event that affects specific groups of a given population at the same time (primary sick persons), but also and especially the ability of the disease to spread quickly to other indefinite groups in the same territory (secondary sick persons). The history of tuberculosis is characterised almost constantly by a phase of infection following exposure that can—sometimes non-sequentially, far from systematically, and in a very limited number of cases— lead to active tuberculosis. This is the only stage during which the bacteria can be transmitted outside the infected person, which prevents any multiple simultaneous occurrences of the disease within a population from being deemed criminally prosecutable epidemics.^19^

This does not change the fact that tuberculosis, while not meeting the requirements to be considered injurious to public safety, can lead in a certain well-defined category of ill subjects to charges of offences against a person, i.e., to the patient’s personal health.^19^

These charges can take the form of personal injury claims independent of the length of the disease’s course, and the charges may vary depending on whether there is full recovery or recovery with permanent sequelae, or if the disease results in the death of one or more affected persons and results in murder charges.

While the crime of murder is easily understood and requires no special explanation, the concept of personal injury deserves a more detailed explanation because it allows further clarification of a distinction existing between clinical and legal language: that of the concept of disease.

## Can Tuberculosis Cause A Prosecutable Disease?

In the medical field, although there is not only one view, in light of the well-known WHO definition,^20^ it is widely accepted that disease is not equivalent to the absence of health.

In practice, all known pathologies, classified by organic and psychiatric criteria, are considered diseases from a clinical point of view.

In legal parlance, the concept of disease is different. It is any deterioration of a progressive nature, anatomical or not, of the body (for example, a trivial excoriation), which can be shown to have resulted in a significant limitation of a bodily function.

This definition is present in substantially all legal systems and is applicable both in criminal and in civil proceedings.

However, it is no coincidence that the concept of disease as understood in criminal law, which in civil proceedings is referred to as disability (temporary=disease, permanent=sequelae), presupposes a condition that has resulted in functional limitations to an organ or the entire organism.^21^

In this context, while it is certainly easy to assign the pathology “tuberculosis” as clinically expressed with its set of symptoms and signs to the realm of disease/disability, with a duration substantially comparable to the observed course and possible outcomes assessed with the aid of specific tables,^21^ an attempt to systematise the nosological entity known as “latent tuberculosis infection” is more complex than it appears.

To try to clarify this point it appears necessary to recall some concepts of purely clinical and anatomical pathology.

The well-known work on pathologic anatomy by Robbins and Cotran emphasises that it is important to differentiate between infection with M. Tuberculosis and the full-blown disease.^22^ Infection is defined as the presence of microorganisms that may or may not cause clinically significant tissue damage (i.e., the disease).

Numerous publications show the substantial difference between the two concepts,^23^,^24^ because tuberculosis infection is substantially the biological condition that follows contact with the mycobacteria and the settling of the bacteria in the body, in the absence of any clinical manifestations of disease.

The term “latent tuberculosis infection” is in turn a further specification of the disease that embraces a wide spectrum of biological conditions^25^ reflecting the natural history of the disease.

As noted previously, not all those who come into contact with tuberculosis bacteria become infected.^9^ In fact, the pathogen may be eliminated altogether^26^ without leaving a trace of its swift passage in the memory of the immune system.^25^ The pathogen, after a settling period, may be still eliminated, but its passage essentially creates immunological memory – an acquired immune response^25^ – thanks to an “immunological scar” that can be detected using Mantoux and/or IGRA (QTF) tests. Additionally the bacteria, though not eliminated, may still be adequately countered and quarantined by the body and contained in a manner that prevents proliferation and more extensive local and systemic damage. This condition has been described as “walled in” and is now referred to as “quiescent infection”.^25^

These stages characterise the anatomo-pathological state usually referred to as “primary complex.” The state is a spectrum of instances of tissue damage^27^ that includes exudative alveolitis (which heals completely), transient Ghon’s complex (which is completely reabsorbed, also usually healing completely), Ghon’s complex with scarring (sometimes including disappearance of caseous necrosis and bacteria), Ghon’s complex leading to fibrosis and calcification (also called Ranke complex, where bacteria are not detected by the microscopy^22^) and Ghon’s complex resulting in a shell of bone tissue containing caseous necrosis residues and Koch bacilli (“walled in”)^27^.

It is therefore appropriate to reiterate that there is not a single pattern of tuberculosis infection, but a number of possibilities ranging from the absence of microorganisms to the presence of a small number of quiescent bacteria, with no signs of disease present, which scientific literature (in the presence of positive immunological tests, most importantly the Mantoux test) has termed “latent tuberculosis infection”.^28^

The fact that a positive immunological test potentially identifies both quiescent infection and complete elimination of the infecting agent resulting in activation of immunological memory (the so-called acquired immune response) justifies the claim that, at present, there is no test that can be designated as the “gold standard” for determining a patient’s exact stage of infection. This disarming assertion is joined by the prevalence of false positives common to any diagnostic method,^29^ as well as the drawbacks specific to tuberculosis tests. One phenomenon is positivity due to laboratory or testing error, which for the QTF test can reach values of 30% of positive results that, spontaneously, i.e., in the absence of potentially confounding factors, convert within three months to a negative result.^30^ Additionally, a positive Mantoux test may be the result of a confounding circumstance, such a previous BCG vaccination or infection by other mycobacteria^28^ and also the observation, for some categories of subjects tested, of the interference of known immunological factors.^31^ These include “chimerism” (the presence of maternal T cells, possibly specific for tuberculosis mycobacterium, in mothers positive to the Mantoux test, which cross the hemo-placental barrier and can circulate in a new born infant for several months or even years) and “trogocytosis” (i.e., the “vampirisation” of foetal/neonatal cells by maternal immune cells specific for the mycobacterium). Both occurrences are potentially responsible for a true positive result, but are descriptive of an immune response temporarily transmitted by the actually sensitised subject to a subject testing positive, but in the absence of exposure (e.g.: mother-infant). This explains why there is no agreement even on the diagnostic criteria for latent tuberculosis infection in the absence of a gold standard test.

This is also the reason why attempts to reach a consensus on the definition of latent tuberculosis infection were hampered by the following conclusions: “tuberculosis” refers to clinically, bacteriologically, histologically and/or radiologically active disease. “Latent infection with *M. tuberculosis*” is usually defined as presumptive infection with *M.* tuberculosis complex, as evidenced by a “positive” tuberculin skin test reaction and/or a positive interferon-γ release assay (IGRA), without any sign of clinically or radiologically manifest disease. However, the biological nature of latent infection with *M. tuberculosis* is controversial. Direct identification of individuals who are latently infected with live *M. tuberculosis,* without active tuberculosis, is currently not possible. The proportion of individuals with a persistently positive immune response against *M. tuberculosis* by tuberculin skin test or IGRA who are truly latently infected with live mycobacteria is unknown. The acronym “LTBI” is commonly used synonymously to describe latent infection with *M. tuberculosis* […]. It is used pragmatically to describe those individuals with a positive adaptive immune response in the tuberculin skin test or in a *M. tuberculosis*-specific IGRA, who are potentially infected with *M. tuberculosis*”.^32^

If, therefore, the term “latent tuberculosis infection” serves to identify those subjects with positive immunological test results but free of clinically active tuberculosis, without determining whether these same individuals are carriers of the walled-in quiescent bacteria (that is, without distinguishing with certainty whether they are infected). Therefore, there can be no doubt that merely detecting this condition cannot in any way meet the conditions for personal injury in the legal sense, for lack of conclusive evidence of its biological assumption: the positive identification of bacteria in the subject testing positive to an immunological test.

Moreover, as stated, the concept of disease in criminal proceedings is proven not only by identifying a state of alteration of the anatomy or of the structure of an organ or tissue but also, and above all, through the necessary presence of a dysfunction of that organ or tissue.

This is why, if diagnostic tools are developed that are capable of detecting the actual presence of dormant *Mycobacterium tuberculosis* in a subject without active tuberculosis^33^,^34^, this might not even be considered sufficient to define this state as a disease in the legal sense.

It is well known that various parts of the human body (skin, respiratory system, gastrointestinal tract) host millions of commensal bacteria, their presence not resulting in any clinically manifest disease. In some circumstances, their physiological presence, for reasons of natural saprophytism, is advantageous. Such is true, for example, in the case of intestinal flora, which improve the absorptive capacity of the gastrointestinal system. In other situations, microorganisms are introduced in large numbers in the human host for therapeutic reasons (for example, with transplantation of microbiota for the treatment of IBD)^35^ or in prophylactic procedures (for example, the numerous types of vaccinations by inoculation with live, albeit attenuated, microorganisms: a prime example is the Calmette-Guerin bacillus for tuberculosis prophylaxis).

Furthermore, because of the same considerations, the immune reaction triggered as a result of contact with the bacteria cannot be considered equivalent to the compromised function necessary for disease in the legally prosecutable sense or for disability under civil law because delayed hypersensitivity is the principal expression of the full functionality of the body’s defences against the tuberculosis mycobacteria. Additionally, this specific immune mechanism – that is, causing a predisposition to quick onset of delayed hypersensitivity — is a technique used to prevent the onset of tuberculosis. Vaccination by injection with attenuated strains of Mycobacterium bovis (a.k.a. the Bacillus Calmette-Guerin or BCG vaccine) attempts to restrict bacillary growth in the event of a new attack by *Mycobacterium tuberculosis*.

Nor, finally, neither can chemoprophylaxis be deemed a criminally prosecutable disease. This is offered to individuals who, inherently or due to exposure, are considered to be particularly at risk of developing the disease. If this were not the case, all chemoprophylaxis procedures that, for example, are performed on healthy subjects who are in transit to places of proven endemic presence of malaria and salmonella for work or pleasure, would be considered criminally prosecutable diseases.

## Tuberculosis and Health Care Work Environments

In a study of point prevalence conducted in a German hospital, the percentage of healthcare workers who tested positive for tuberculin was 34.8%.^36^ In France, prevalence was calculated at 33%, while in Portugal this figure rose to 45%.^37^

The phenomenon of latent tuberculosis infection is widely represented in healthcare workers even when they operate in countries with a low incidence of the disease.

Even if, as mentioned, the probability that immunological test positivity corresponds to an actual state of quiescent infection is low, and even if the likelihood that subjects with quiescent infection actually develop clinically manifest tuberculosis during the course of their lives is even more remote, the data suggest that monitoring the trend is desirable. Doing so also serves to protect the health of workers who, as carriers, are at increased risk of developing full-blown tuberculosis, particularly if re-exposed to other sources of infection during the course of their activities.

Furthermore, European Community regulations on employee health contain other references requiring the health and safety of third parties present in the workplace who might suffer from the consequences of actions or omissions of workers.

It therefore seems logical to infer that, by virtue of these regulations, medical opinions on fitness for specific jobs– which are a workplace safety measure–can also legitimately contain provisions for the protection of other co-workers or third parties.

If this is a desirable recommendation, for example, additional personal protective equipment, we must also ask whether this also applies to testing for possible carriers of infectious diseases such as HIV, HCV or TB.

It must be recognised that the protection of health has collective implications arising from the need to combat forms of epidemic morbidity and to ensure general public health. This requires a perspective that takes into account the interest of the community and can sometimes lead to restriction of individual freedoms. To safeguard individuals, however, the constitutional laws of modern countries, quite impervious to discontent, concerns and irrational fears of ordinary people, usually include a saving clause: cases and forms of mandatory health treatment must be specified in advance by law, and secondary provisions can always be added. A legal provision, however, is not enough. In formulating laws, legislators must take into account the inviolable boundary of respect for the human person.

It would be difficult even for the strictest litigator to identify those special cases within the healthcare sector for which the need of the community is greater than that of the individual, such that *a priori* use of indiscriminate mass health checks for all people or categories of persons is justified; such a measure is obviously disproportionate to the need for protection of public health. More often, health assessments are limited both in who can be compelled to submit to them as a condition of carrying out a particular activity, as well as in the content of assessments: findings in each case are functionally related to performance of those specific activities and reserved for those who are, or intend to be, involved.

Therefore, that in order to protect the health of the community from the risk tuberculosis transmission among and by healthcare workers, especially those already identified as carriers of latent tuberculosis infection and therefore not contagious, but at risk of developing tuberculosis disease (and thus infectivity), the decision to monitor frequency and evolution is usually left to the clinical evaluation of an institutional medical professional responsible for monitoring the health of workers. It is the responsibility of this professional figure to determine the type and frequency of tests to be offered to the employee once potential exposure to occupational risk is assessed.

In respect for individual freedom and health of the individual, it would be appropriate that, in the absence of known risk factors, these assessments not be mandatory; refusal to undergo them by employees might nevertheless affect eligibility for a position.

However, it is clear that particularly in the case of tuberculosis, ineligibility can be decided only when, during health assessments, even when specific results are not present because a worker refuses testing, the examiner has reason to suspect the presence of a pathological condition, i.e., an actual disease that merits therapeutic and behavioural measures (including temporary suspension from work) to protect the health of the worker. These measures are even more necessary in the presence of bacilliferous, or infectious, states, capable of spreading via human contact and, as such, are a source of real danger for the health of other persons (colleagues or people outside the workplace).

Very different is the case in which the worker is known to have tested positive to immunological tests. In such cases one can assume that certain environments may represent an additional biological risk to his/her health, which justifies restrictive protective measures such as partial ineligibility of the worker for certain activities involving such risk.

However, such measures may appear disproportionate, especially when the employee agrees to submit to periodic assessments and post-exposure testing, because the monitoring of subjects positive to these tests could be considered sufficient to ensure the health of workers and other persons.

This is, moreover, the reason for which international guidelines on the management of the screening of healthcare workers exposed in low-risk healthcare facilities suggest that a healthcare worker who undergoes a Mantoux test “should not repeat screening until a new exposure occurs”^38^. This establishes that a chest radiograph examination should be performed for healthcare professionals whose Mantoux test confirms tuberculosis infection or who present conversion (observed increase of at least 10 mm of maximum diameter of the skin reaction in a period of two years), or those who test positive to another immunological test (QTF) or who are undergoing treatment for latent tuberculosis infection. The radiograph serves to rule out active-phase tuberculosis; in such circumstances, a negative test is a sufficient condition to presume the worker is healthy, and repeat testing is “not needed unless the patient has symptoms or signs of TB disease or unless ordered by a physician for a specific diagnostic examination”^38^.

In the event an employee is suffering from tuberculosis, the employee becomes temporarily unfit to work and, in countries with a social security system, is eligible for benefits and financial support not payable for mere latent tuberculosis infection.

## Tuberculosis and Obligatory Medical Treatment

The possibility that a patient with pulmonary tuberculosis can spread of tuberculosis in the population by infected patients is currently countered using two treatment measures. One is hygienic and behavioural in nature and consists of isolation in the initial stage. The other measure is pharmacological and consists of initiating an appropriate therapy regimen.

These two solutions, especially the former, take on greater importance in patients with forms of multidrug-resistant tuberculosis.

No problem arises when the two treatments are accepted by the patient. This changes if patients reject such treatment solutions.

In such cases, in several legal systems, there is the possibility of forced, mandatory treatment to protect public health and prevent the spread of pathogens, especially those that are multidrug-resistant.

Obviously this is a measure governed by law, which provides for the involvement of guarantors other than the doctor who suggest or prescribe mandatory treatment or isolation. These guarantors might be, for example, other doctors, the mayor of the city where the patient resides, or probate judges.

The use of guarantors serves to balance the interests of the public with those of the patient (which mandatory treatment can subject to pressure).

One must consider that, to balance the needs of the individual with those of public health, mandatory treatment, even if codified, cannot compromise the dignity of the patient. The duration and extent of treatment must be proportionate to the actual danger present.

Therefore, mandatory isolation, lasting longer than 15 days, of a patient fully adherent to pharmacotherapy and a carrier of non-multidrug-resistant TB,^28^ would constitute a blatant violation of the dignity of the patient and could result in criminal charges and possibly compensation of damages in civil proceedings.

On the other hand, failure by the physician to recognise the conditions requiring measures to cure and contain the disease could lead to professional liability in the event of deterioration of the patient’s health or spread of the disease to individuals entering into contact with the patient.

## Conclusions

Tuberculosis is a disease that has accompanied the evolution of man from the earliest social structures, similar to other infectious diseases famous for their historical lethality (smallpox, influenza, malaria, plague, measles and cholera).^39^

Tuberculosis, as well as other diseases mentioned above, were decisive factors in the course of human history.

When the transcontinental railroad was built in Canada circa 1880, the Indians of Saskatchewan met both the whites and the Koch bacillus, and began to die of tuberculosis at the frightening rate of 9% per year.^39^

Not only, along with its sister diseases, tuberculosis can even determine the outcome of military conflicts, with the winners not decided by strategic and military preparation, but rather according to who had historically been more immunised.

It is inevitable that similar diseases may leave their mark not only on the collective memory in the form of ancestral fears but also in the regulatory systems that govern the current societies descended from the initial aggregations of our predecessors at the beginning of their emergence and diffusion.

Based on these experiences and, of course, are affected by the plasticity of the human organism and its ability to adapt over time and coexist with these illnesses through the ages.

In this respect, tuberculosis can be seen as a paradigm: because knowledge of its natural history has been established over centuries of observation, it has been possible to arrive at a coherent formulation of the crime of dissemination of an epidemic. The need to protect public safety limits the crime to contagious infectious diseases that, unlike tuberculosis, are able to spread rapidly and simultaneously in large sections of a target population, and therefore constitute a serious threat of equally rapid transmission to subjects initially spared.

Tuberculosis can be viewed as a paradigm in a different respect. In countries with a low incidence of the disease and in certain working environments, such as within disease centres, the prevalence of latent tuberculosis infection – as mentioned, an ill-defined corollary of tuberculosis—has now reached significant percentages, to the point of real social concern that, uncontrolled, health care workers may one day become carriers of the pathogen exposing the most vulnerable strata of the remaining population: patients admitted to these centres for other reasons.

It is obvious that monitoring of the phenomenon is necessary, but it is equally clear that such monitoring systems inevitably involve a decrease in the freedom and individual aspirations of workers, particularly in the case of controls with overly restrictive measures that force workers to stop their chosen profession in the name of protecting public health, even if well-intentioned.

Again, tuberculosis has been problematic in the historical development of our societies and does so in typical provocative style, posing uncomfortable questions to various legislators and their interpreters: is it legitimate to sacrifice the freedom of the professional on the altar of the precautionary principle, supposedly in place to protect public health?

On closer inspection it is the agonising dilemma that runs through the history of liberal states: to reconcile the requirements for protecting the freedom and interests of both the individual and the group.

The balance of interests is an unresolved dilemma that this ancient infectious disease (together with similar diseases) forces us to address even today, particularly because of its impact in the workplace and in social life.
